# Photodynamic therapy remodels cutaneous tumor immunity: innate priming, suppressive feedback, and translational opportunities

**DOI:** 10.3389/fimmu.2026.1931149

**Published:** 2026-09-08

**Authors:** Yang Chen, Wen Luo, Jiejie Lu, Weiwei Wu

**Affiliations:** 1 Department of Dermatology, The Fifth People’s Hospital of Hainan Province, Haikou, China; 2 Hainan Medical University, Haikou, Hainan, China; 3 Department of Dermatology, Affiliated Dermatology Hospital of Hainan Medical University, Haikou, China

**Keywords:** cutaneous neoplasms, immune microenvironment (IME), immunogenic cell death (ICD), innate immune priming, photodynamic therapy (PDT), suppressive feedback, translational opportunities

## Abstract

Photodynamic therapy (PDT) is an established local treatment for actinic keratoses, Bowen disease, and selected low-risk basal cell carcinomas, but its potential to reshape antitumor immunity may extend its value beyond direct phototoxicity. We conducted a narrative synthesis of clinical, translational, and mechanistic evidence, supplemented by targeted searches through August 2026, and appraised major findings using a three-level evidence-maturity framework. Current evidence supports a temporally organized model in which PDT-induced reactive oxygen species promote tumor and microvascular injury, immunogenic cell death, damage-associated molecular pattern release, tumor-antigen exposure, innate immune-cell recruitment, dendritic-cell activation, and subsequent T-cell priming. This early immune-priming phase may be followed by suppressive feedback involving hypoxia, tumor-associated macrophages, regulatory T cells, myeloid-derived suppressor cells, PD-1/PD-L1 signaling, and IL-10/TGF-β. Evidence maturity and translational relevance vary across cutaneous neoplasms: clinical use is best established for actinic keratoses, Bowen disease, and superficial or selected low-risk basal cell carcinomas, whereas PDT-based immunotherapy combinations in invasive cutaneous squamous cell carcinoma and melanoma remain predominantly preclinical or exploratory. In cSCC models, ALA-PDT combined with PD-L1 blockade improved control of primary and distant tumors, providing preclinical support for further translational evaluation of checkpoint-based combinations. Preclinical timing studies further suggest that combination efficacy may depend on aligning checkpoint blockade with the post-PDT T-cell activation window. Photosensitizer localization, fluence, irradiance, oxygenation, and treatment schedule may likewise determine whether PDT favors immunogenic priming or excessive tissue injury and suppressive rebound. These findings position PDT as a parameter-tunable local immune-priming platform whose clinical translation should integrate indication-specific selection, treatment-parameter optimization, temporal immune monitoring, rationally timed combination therapy, and prospective clinical validation.

## 1 Introduction

Photodynamic therapy (PDT) is a local treatment modality that requires a photosensitizer, light of an appropriate wavelength, and molecular oxygen. Following preferential accumulation in target tissues, photosensitizers are activated by light and generate reactive oxygen species (ROS), which can induce tumor-cell injury, microvascular damage or occlusion, local inflammation, and ultimately tumor-cell death (1). In cutaneous oncology, PDT has been used primarily for superficial and locally accessible lesions, particularly actinic keratosis, Bowen disease, superficial basal cell carcinoma (BCC), and selected low-risk thin or nodular BCC. By contrast, in invasive cutaneous squamous cell carcinoma (cSCC), high-risk BCC, and melanoma, PDT is generally not considered a standard curative modality and should not replace stage- and risk-adapted standard treatments (2).

Recent studies have expanded the understanding of PDT beyond direct phototoxic killing. Basic and translational evidence indicates that PDT can induce immunogenic cell death (ICD) through ROS-dependent cellular stress, accompanied by cell-surface exposure of calreticulin (CRT), ATP release, HMGB1 release, heat-shock protein (HSP) exposure, and activation of type I interferon-related signaling. These damage-associated molecular patterns (DAMPs) can function as danger signals and endogenous immunoadjuvants, thereby promoting antigen uptake, dendritic-cell maturation, antigen cross-presentation, and subsequent T-cell priming. Under specific conditions, PDT may therefore act not only as a local cytotoxic treatment but also as a local immune-priming modality that links local tumor injury to broader antitumor immune responses (3). Importantly, consensus guidelines define ICD by the capacity of dying cells to elicit an adaptive immune response against dead-cell-associated antigens, rather than by the occurrence of cell death alone (4).

**Figure 1:**
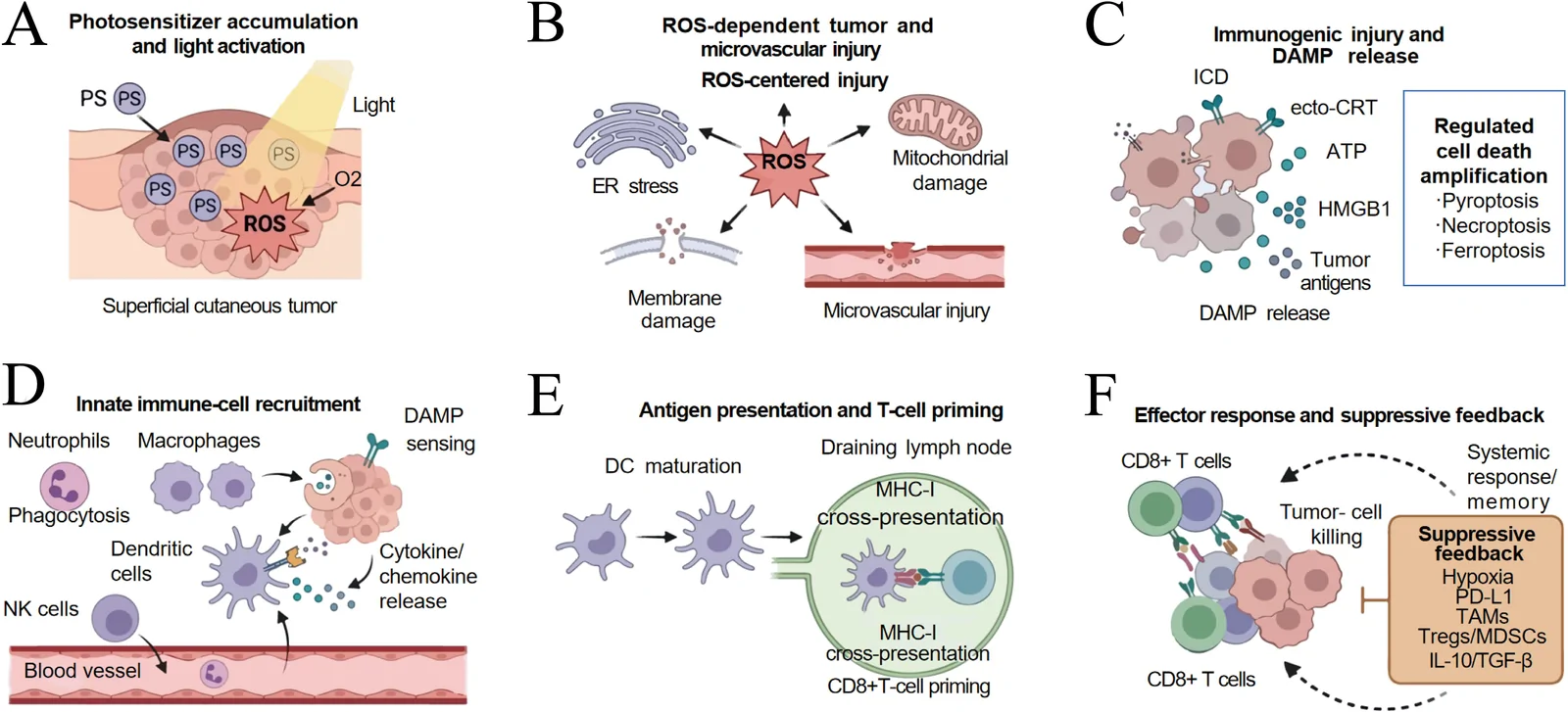
PDT-induced immunogenic injury and immune remodeling in cutaneous neoplasms. **(A)** Photosensitizer accumulation in superficial cutaneous tumor lesions followed by light irradiation generates reactive oxygen species (ROS) in the presence of tissue oxygen. **(B)** ROS-dependent photodynamic stress induces tumor-cell injury and microvascular damage, including endoplasmic reticulum stress, mitochondrial injury, membrane damage, and vascular injury. **(C)** These events promote immunogenic cell death (ICD), damage-associated molecular pattern (DAMP) exposure or release, and tumor-antigen release. Representative ICD-associated signals include ecto-calreticulin (ecto-CRT), ATP, and HMGB1. Under selected conditions, pyroptosis, necroptosis, and ferroptosis may further amplify inflammatory danger signaling. **(D)** DAMPs and tumor antigens recruit innate immune cells, including neutrophils, macrophages, dendritic cells (DCs), and natural killer (NK) cells, and support phagocytosis, antigen processing, cytokine/chemokine release, and DC activation. **(E)** Mature DCs migrate to draining lymph nodes, mediate MHC-I cross-presentation, and prime CD8^+^ T-cell responses. **(F)** The downstream effector phase may promote CD8^+^ T-cell infiltration, tumor-cell killing, systemic immune activation, and immune memory, whereas hypoxia, PD-L1, tumor-associated macrophages (TAMs), regulatory T cells (Tregs), myeloid-derived suppressor cells (MDSCs), IL-10, and TGF-β may contribute to suppressive feedback after PDT.

However, PDT-induced immune activation does not necessarily translate into durable and systemic antitumor immunity. Within the tumor microenvironment, hypoxia, immunosuppressive tumor-associated macrophages (TAMs), regulatory T cells (Tregs), myeloid-derived suppressor cells (MDSCs), the PD-1/PD-L1 axis, and suppressive cytokines such as IL-10 and TGF-β may collectively restrict the persistence and amplification of post-PDT immune responses (5). Some experimental studies further suggest that high-light-dose PDT may elicit a biphasic immune response, characterized by early immune activation followed by late-stage immunosuppressive rebound. Thus, the effect of PDT on the tumor immune microenvironment is better interpreted as a dynamic balance between immune activation and compensatory suppression, rather than as a unidirectional immune-enhancing process. In particular, TGF-β1-mediated late immunosuppression after high-light-dose PDT has been supported by experimental evidence (6). The major sequence of PDT-induced immunogenic injury, innate immune-cell recruitment, antigen presentation and suppressive feedback is summarized in **Figure 1**.

Existing reviews have extensively discussed the general mechanisms by which PDT induces ICD and antitumor immunity. However, cutaneous tumor-specific evidence stratification has received comparatively limited attention (3, 7). In particular, the clinical boundaries of PDT across different cutaneous tumor subtypes, the temporal roles of innate immune cells, the relationship between immunosuppressive reprogramming and feedback regulation, and the optimal timing of PDT combined with immune checkpoint blockade have not yet been integrated within a cutaneous tumor-specific framework. Unlike previous reviews that have mainly focused on PDT-mediated phototoxic tumor destruction or general mechanisms of immunogenic cell death, this review emphasizes cutaneous tumor-specific evidence stratification and further integrates innate immune-cell recruitment, antigen presentation, suppressive feedback, treatment parameter dependency, and the translational boundaries of PDT-based combinations with immune checkpoint blockade and related immunotherapeutic strategies.

To avoid directly extrapolating pan-cancer PDT mechanisms, engineered photosensitizer studies or preclinical nanoplatform-derived findings to clinical cutaneous tumors, this review uses an evidence-maturity framework that links mechanistic interpretation with clinical translation. Within this framework, PDT-related evidence is stratified into established clinical applications, preclinical or translational immune-sensitizing strategies, and conceptual exploratory directions. This approach helps distinguish clinical indications from mechanistic plausibility and hypothesis-generating nanoplatform-based findings (8).

Methods: literature search, study selection and evidence appraisal

This article was designed as a narrative review rather than a systematic review or meta-analysis. Literature searches were conducted in PubMed, Web of Science Core Collection and Google Scholar from database inception to May 2026. The search strategy combined three thematic blocks: PDT-related terms, cutaneous tumor-related terms and immune mechanism-related terms. The core Boolean search string was: (“photodynamic therapy” OR PDT OR “photodynamic treatment” OR photosensitizer OR “5-aminolevulinic acid” OR ALA OR “methyl aminolevulinate” OR MAL) AND (“cutaneous tumor” OR “cutaneous neoplasm” OR “skin cancer” OR “actinic keratosis” OR “Bowen disease” OR “squamous cell carcinoma in situ” OR “basal cell carcinoma” OR “cutaneous squamous cell carcinoma” OR cSCC OR melanoma) AND (“immune microenvironment” OR “tumor microenvironment” OR “immunogenic cell death” OR “damage-associated molecular patterns” OR DAMPs OR “innate immunity” OR neutrophil OR macrophage OR “dendritic cell” OR “natural killer cell” OR “T cell” OR “PD-1” OR “PD-L1” OR Treg OR MDSC OR “IL-10” OR “TGF-β” OR hypoxia OR STING OR TLR OR pyroptosis OR necroptosis OR ferroptosis OR cuproptosis OR nanoplatform OR immunotherapy). Field tags and synonyms were adapted according to the requirements of each database, and reference lists of relevant guidelines, clinical studies and review articles were manually screened. During revision, targeted supplementary searches were performed through August 2026 to identify additional evidence on mast-cell involvement, melanoma-specific PDT resistance, and clinical PDT–immunotherapy combinations.

Eligible publications included guidelines, consensus statements, clinical studies, translational studies, animal experiments, ex vivo studies, *in vitro* mechanistic studies and high-quality reviews related to PDT, cutaneous tumors or cutaneous precancerous lesions. Studies were included if they addressed PDT-mediated antitumor effects, immunogenic cell death, DAMP release, innate immune-cell recruitment, antigen presentation, immunosuppressive feedback, treatment parameters or immunotherapy combinations. English-language publications from January 2000 to May 2026 were prioritized, while earlier landmark studies were retained when they provided foundational evidence for PDT immunology, clinical indications or treatment-parameter concepts. Exclusion criteria included studies unrelated to PDT or cutaneous tumor immunity, studies focused only on non-neoplastic skin diseases or non-PDT interventions, purely materials-oriented reports without mechanistic or translational relevance, conference abstracts, news reports, brief commentaries, inaccessible records, duplicates and highly overlapping publications. Non-cutaneous tumor models, pan-cancer PDT studies and engineered nanoplatform studies were not excluded *a priori*, but were used only for mechanistic extrapolation or exploratory discussion rather than as direct evidence for clinical cutaneous tumor practice.

Records were screened first by title and abstract and then by full text for thematic relevance, evidence maturity and mechanistic relevance. The initial screening was performed by one reviewer; key included studies, boundary cases and references supporting major claims were checked by a second reviewer, with disagreements resolved by discussion. Because this was not a systematic review, no quantitative synthesis or PRISMA flow diagram was performed. The evidentiary weight of included sources was appraised narratively, using SANRA-informed criteria and the evidence-maturity framework adopted in this review (8). The operational definitions of the three evidence-maturity levels are provided below and were used to guide the interpretation of major claims and the construction of **Table 1**.

**Table 1:** Clinical positioning, evidence maturity and immunological implications of PDT across cutaneous neoplasms and precursor lesions.

Tumor type	Clinical positioning of PDT	Common PDT strategies	Major limitations	Immunological implications for combination therapy	Evidence maturity
AK, Bowen’s disease and cSCC spectrum	Established use is mainly in AK and Bowen’s disease; PDT is not a substitute for curative treatment in invasive or high-risk cSCC (2, 41).	AK: ALA/MAL-PDT or daylight PDT. Bowen’s disease: ALA- or MAL-mediated red-light PDT (2).	Limited efficacy for thick, hyperkeratotic, suspected invasive or high-risk lesions; histological confirmation is required when invasion is suspected (2, 41).	Mainly supports local treatment and field cancerization management; ALA-PDT plus PD-L1 blockade provides preclinical immune-priming evidence in cSCC (35).	Level 1 for AK/Bowen’s disease clinical use; Level 2 for cSCC immune-combination evidence.
BCC	Suitable for superficial BCC and selected low-risk thin nodular BCC when tissue preservation or cosmesis is prioritized (2, 42).	ALA/MAL-PDT with red light around 630 nm; MAL-PDT commonly uses two sessions separated by 1 week (2, 42).	Long-term local control is inferior to surgery, especially for thicker, recurrent, high-risk-site or aggressive subtypes (2, 42).	PDT may enhance Hip1-related systemic immune reactivity, but recurrence reduction is unproven; advanced BCC remains managed mainly with HHI and selected PD-1 blockade (18, 42).	Level 1 for superficial/selected low-risk BCC clinical use; Level 2 for Hip1-related systemic immune reactivity.
Melanoma	Not a standard primary treatment; mainly an experimental local immune-priming or combination strategy (47, 48, 52).	Modified photosensitizers, nanodelivery, near-infrared/two-photon strategies and immunotherapy combinations (48, 52).	Limited by melanin-related optical interference, antioxidant defenses, lesion depth, heterogeneity and insufficient light penetration (48).	May induce immunogenic injury, antigen release and inflammatory remodeling, supporting preclinical combinations with vaccines, adjuvants or checkpoint inhibitors (52).	No established Level 1 role for conventional PDT; Level 2–3 for immune-combination and nanoplatform strategies.

Evidence-maturity framework

To align mechanistic interpretation with translational relevance, major claims in this review were interpreted according to a three-level evidence-maturity framework. Level 1 evidence was defined as established clinical evidence, including clinical guidelines, consensus statements, randomized or comparative clinical studies, and human studies that directly inform clinical indications, treatment positioning or patient management. In this review, Level 1 evidence mainly supports the established clinical use of topical PDT in actinic keratosis, Bowen’s disease, superficial BCC and selected low-risk thin BCC, as well as the clinical boundaries of PDT in invasive cSCC, high-risk BCC and melanoma.

Level 2 evidence was defined as preclinical or translational evidence, including animal models, ex vivo studies, human tissue- or blood-based immune observations, and cutaneous tumor-relevant mechanistic studies. This level was used for claims linking PDT to immune-cell recruitment, dendritic-cell activation, antigen presentation, macrophage or neutrophil responses, NK-cell-associated signals, and combination strategies such as ALA-PDT with PD-L1 blockade in cSCC models. Such evidence can support biological plausibility and translational rationale, but was not treated as proof of clinical efficacy unless supported by human clinical data.

Level 3 evidence was defined as conceptual or exploratory evidence, including *in vitro* experiments, non-cutaneous tumor models, pan-cancer PDT studies, engineered photosensitizer or nanoplatform studies, and mechanism-oriented studies without direct cutaneous tumor validation. This category was applied to exploratory mechanisms such as pyroptosis, necroptosis, ferroptosis, cuproptosis, some nanoplatform-based immune-sensitizing strategies, and most PDT-based immunotherapy concepts in melanoma. These findings were interpreted as hypothesis-generating evidence rather than as direct support for clinical practice in cutaneous tumors.

This framework was applied throughout the review to avoid treating clinical indications, preclinical immune mechanisms and exploratory nanoplatform-based findings as equivalent forms of evidence. It also guided the interpretation of **Table 1**, in which clinical positioning, immunological implications and translational boundaries were separated according to evidence maturity.

## 2 Core mechanisms by which PDT initiates immune remodeling: ROS, ICD, DAMPs, and antigen exposure

PDT-mediated remodeling of the immune microenvironment does not begin with the immediate accumulation of effector T cells. Rather, it starts when tumor cells exposed to photodynamic stress die in a way that can be detected and handled by the immune system. Immunogenic cell death (ICD) refers to a form of regulated cell death in which dying tumor cells release tumor antigens and expose damage-associated molecular patterns (DAMPs), thereby promoting adaptive immune responses against antigens derived from dying cells. Unlike non-immunogenic cell death, ICD is not defined by cell death alone, but by whether dying cells provide both antigenic and adjuvant signals that are sufficient to initiate an immune response (4).

During PDT, light-activated photosensitizers generate ROS and trigger cellular stress responses, including endoplasmic reticulum stress, autophagy, mitochondrial injury, and membrane permeabilization. These processes promote the emission of ICD-associated DAMPs and signals, such as cell-surface CRT exposure, extracellular ATP release, HMGB1 release, ANXA1 mobilization, and type I interferon-related signaling. Functionally, CRT acts as an “eat-me” signal for phagocytes, ATP serves as a “find-me” signal that recruits and activates antigen-presenting cells, and HMGB1 contributes to dendritic-cell maturation and antigen processing. Type I interferon signaling may further strengthen the link between dendritic-cell activation and tumor-specific CD8^+^ T-cell priming. Thus, PDT-induced DAMP emission can convert otherwise silent tumor-cell death into immune-relevant signals that are captured, processed, and presented by dendritic cells. Although the general framework of PDT-induced ICD and DAMP exposure is well supported, the magnitude and timing of these events in specific cutaneous tumor settings remain insufficiently defined (3, 9).

From an evidence-chain perspective, CRT exposure, ATP release, HMGB1 release, ANXA1 release, and activation of type I interferon-related signaling can be regarded as early pharmacodynamic signals of PDT-induced immunogenic injury. Their functional translation extends beyond DAMP exposure itself and depends on a sequential process involving antigen release from dying tumor cells, APC uptake, dendritic-cell maturation and cross-presentation, T-cell priming in draining lymph nodes, and the subsequent trafficking of effector T cells back to tumor lesions (4). In cutaneous neoplasms, the strength and completeness of this evidence chain may vary according to tumor type, PDT parameters, lesion depth, and subsequent suppressive immune feedback (9). Accordingly, the immunological relevance of PDT-induced ICD should be interpreted within the continuum of innate immune-cell recruitment, antigen presentation, and T-cell priming.

## 3 Innate immune recruitment, antigen presentation, and T-Cell priming after PDT

After PDT, innate immune activation unfolds as a time-ordered cascade that connects local inflammatory damage with antigen presentation. Early after PDT, ROS-mediated injury, DAMP exposure, and cytokine release create chemotactic gradients that recruit neutrophils and other myeloid cells to the treated lesion (10). Mast cells may also participate in early inflammatory and vascular remodeling after PDT, although their functional role in cutaneous tumors remains less well characterized (11, 12). As this acute inflammatory phase progresses, monocytes and macrophages help clear dying cells and process released antigens, while dendritic cells capture tumor-associated material and migrate to draining lymph nodes for cross-presentation (13). NK cells may add an early cytotoxic component before the DC–T-cell axis is fully established and can further shape local immunity through IFN-γ and related cytokine networks (14). In this context, the key question is not simply whether immune cells enter the lesion after PDT, but how their recruitment is organized over time and how efficiently it is coupled to antigen presentation and T-cell priming.

### 3.1 Rapid recruitment and functions of neutrophils

Neutrophils are among the first leukocytes to accumulate in PDT-treated lesions. PDT-induced tissue injury is accompanied by increased levels of inflammatory cytokines and chemokines, including IL-6 and neutrophil-attracting chemokines such as KC/CXCL1 and MIP-2/CXCL2, which promote early neutrophil influx into the treated tumor site (15). Once recruited, neutrophils can contribute to tumor-debris clearance through degranulation, protease release, ROS generation and, in broader inflammatory settings, neutrophil extracellular trap formation. They may also shape subsequent immune recruitment by releasing chemokines that attract dendritic cells, monocytes and T cells (10, 16). In cutaneous tumor PDT, neutrophils are therefore best placed within the early inflammatory-remodeling phase: they amplify acute tissue injury and immune-cell entry, but their contribution to durable antitumor immunity depends on regimen intensity, the extent of tissue damage and the post-treatment time window.

### 3.2 Macrophage reprogramming: phagocytosis, inflammatory shaping, and suppressive rebound

As the early neutrophil-dominant phase evolves, macrophages become central to the transition from inflammatory clearance to immune regulation. PDT-induced ROS generation and DAMP release can push local macrophages toward phagocytic and inflammatory states, increasing uptake of dying tumor cells, production of pro-inflammatory mediators and engagement with antigen-processing pathways. Pattern-recognition, interferon-related and inflammasome-associated signals are likely to participate in this response, although their macrophage-specific roles in cutaneous tumor PDT remain incompletely mapped (13). This inflammatory program is temporally constrained. During tissue repair, local hypoxia together with IL-10, TGF-β and other repair-associated cues can redirect macrophages toward suppressive or reparative TAM-like states (17). Macrophages therefore occupy a dual position after PDT, linking early innate activation and antigen handling with the later re-establishment of an immunosuppressive microenvironment.

### 3.3 Dendritic-cell maturation and antigen presentation

Dendritic cells (DCs) are a key bridge through which PDT may convert a local treatment into an immunomodulatory intervention. After PDT, tumor-associated antigens and DAMPs released from dying tumor cells can promote antigen uptake by DCs, upregulation of MHC molecules and costimulatory molecules such as CD80 and CD86, and enhanced antigen cross-presentation. Mature DCs can then migrate to draining lymph nodes, where they prime tumor-specific CD8^+^ T-cell responses and extend the immunological impact of local photodynamic injury beyond immediate cytotoxicity. In cutaneous tumors, increased peripheral immune reactivity against the BCC-associated antigen Hip1 after PDT provides clinically relevant evidence that this antigen-presentation axis can be engaged in patients with BCC (13, 18). Evidence in other cutaneous tumor subtypes is less direct, making tumor type and treatment context important when interpreting the DC–T-cell consequences of PDT.

### 3.4 Activation of natural killer cells and tumor clearance

NK cells may contribute an early cytotoxic component before fully developed T-cell responses emerge. PDT-induced inflammation and cellular stress can increase tumor-cell susceptibility to NK-cell recognition, while IL-12, type I interferons and other inflammatory cytokines derived from DCs and macrophages may further enhance NK-cell activation. In cSCC-related studies, increased CD56 expression after PDT indicates that NK-cell-associated immune signals may be strengthened during early post-treatment immune remodeling (13, 19).

### 3.5 Mast cells as an underexplored component of post-PDT inflammation

Mast cells represent a less well characterized component of the inflammatory response after PDT. In murine SCCVII tumors, Photofrin-PDT induced a rapid increase in intratumoral mast cells following the initial neutrophil influx (12). In patients with superficial BCC, serial biopsies after ALA-PDT showed a modest increase in mast-cell numbers over 72 h, together with time-dependent changes in neutrophils, macrophages and lymphocytes (11). In a rat model of oral premalignant lesions, 5-ALA-PDT increased mast-cell degranulation and was associated with changes in microvessel density, raising the possibility that mast-cell mediators participate in inflammatory and vascular remodeling after PDT (20). However, the available evidence remains limited by small human datasets and reliance on preclinical or non-cutaneous models, while functional evidence from mast-cell depletion or blockade studies in cutaneous tumors appears to be lacking. Mast cells should therefore be regarded as plausible but incompletely defined modulators of post-PDT inflammation rather than established drivers of PDT-induced immunosuppression.

Taken together, PDT-induced innate immune recruitment is not a single-cell-type event but a temporally organized sequence. Neutrophils initiate acute inflammatory amplification and debris clearance; macrophages integrate phagocytosis, inflammatory shaping and later suppressive reprogramming; DCs link antigen capture to cross-presentation and CD8+ T-cell priming; and NK cells contribute an early cytotoxic and cytokine-mediated component. Mast cells may represent an additional component of early inflammatory and vascular remodeling after PDT, although their functional contribution in cutaneous tumors remains poorly defined. Available evidence therefore supports coordinated, time-dependent remodeling involving multiple innate immune populations rather than dominance by any single immune-cell subset. Better defining the kinetics and functional interactions of these populations, including the underexplored mast-cell compartment, in cutaneous tumor-specific models will be important for optimizing PDT regimens that promote immune priming while limiting delayed suppressive rebound.

## 4 PDT-induced regulated cell death and amplification of immunogenic consequences

Within the evidence-maturity framework used in this review, PDT-induced ICD and DAMP exposure represent a relatively well-supported mechanistic axis, although cutaneous tumor-specific clinical validation remains incomplete. By contrast, pyroptosis, necroptosis and ferroptosis are better interpreted as Level 2–3 immunogenic amplification mechanisms, whereas cuproptosis remains largely within Level 3 materials-based and exploratory directions (3, 21, 22).

Pyroptosis is a lytic and inflammatory form of cell death mediated by gasdermin family proteins (23). In some tumor models, PDT has been reported to induce pyroptosis through pathways such as caspase-3/GSDME or caspase-1/GSDMD, leading to membrane pore formation, cell swelling and rupture, and the release of DAMPs and inflammatory mediators. Because pyroptosis releases intracellular contents in an inflammatory manner, it can intensify local danger signaling after PDT, promote myeloid-cell recruitment and indirectly support DC maturation (22, 24). In cutaneous tumor PDT, this mechanism currently functions best as an immunogenic amplification pathway that may operate under specific treatment conditions, rather than as a consistently established driver of clinical response.

Necroptosis is a regulated necrotic cell-death pathway governed by the RIPK1/RIPK3/MLKL axis. It culminates in plasma-membrane rupture and the release of DAMPs and other immunostimulatory molecules. Under conditions of impaired apoptosis or intense cellular stress, PDT may shift tumor cells toward necroptotic death, increasing antigen release and inflammatory signaling (22, 25). For cutaneous tumors, however, direct evidence connecting necroptosis to post-PDT immune remodeling remains relatively sparse. At present, necroptosis provides a complementary mechanism within the broader spectrum of PDT-associated immunogenic cell death, especially in settings where apoptosis is insufficient to explain the observed inflammatory response (22).

Ferroptosis is characterized by iron-dependent lipid peroxidation and oxidative membrane-lipid damage (26). Because PDT is intrinsically dependent on ROS generation, it has a strong biological link to lipid peroxidation and ferroptosis. Ferroptosis may participate in post-PDT immune microenvironmental changes by releasing lipid peroxidation-derived signals, enhancing inflammatory responses, and altering the immunogenicity of tumor cells (27). However, current evidence is largely derived from non-cutaneous tumor models or engineered nano-photosensitizer platforms, and is not yet sufficient to establish ferroptosis as a consistent axis of immune effects in clinical PDT for cutaneous tumors.

Cuproptosis, a copper-dependent regulated cell-death mechanism linked to lipoylated TCA-cycle proteins, remains an exploratory direction in PDT-related tumor research (28). Current evidence linking cuproptosis to PDT is dominated by engineered nanoplatform and preclinical studies, with only limited cutaneous tumor-specific validation; it should therefore remain Level 3 hypothesis-generating evidence rather than be weighted alongside better-supported PDT-associated cell-death pathways (21).

Across cutaneous neoplasms, the available evidence does not support a single dominant regulated cell-death pathway after PDT. In AK, human biopsy studies demonstrate apoptosis within 24 h together with progressive epidermal necrosis, whereas superficial BCC shows a clear time-dependent apoptotic response after ALA-PDT (11, 29). In cSCC, modified ALA-PDT has been shown to trigger ROS–JNK–NLRP3-dependent pyroptosis in human cSCC cell lines, but this remains preclinical evidence rather than an established clinical death program (30). Melanoma data are likewise predominantly experimental: conventional PDT can induce apoptosis at moderate phototoxic stress and necrosis at higher levels of injury (31). By contrast, ferroptosis has mainly been demonstrated using engineered photoactive platforms in melanoma models (32). Thus, apoptosis currently has the strongest direct human support in AK and BCC, whereas pyroptosis in cSCC and ferroptosis in melanoma represent mechanistically relevant but less clinically mature extensions of PDT-associated cell death.

## 5 PDT-mediated disruption and rebound of the immunosuppressive microenvironment

The effect of PDT on the immunosuppressive tumor microenvironment should not be understood simply as a unidirectional relief of immune suppression. More precisely, the post-PDT immune microenvironment may undergo a biphasic process. In the early phase, ROS generation, DAMP exposure, inflammatory cytokine release, and tumor-antigen liberation drive innate immune activation and antigen presentation (7); In the later phase, however, hypoxia, tissue-repair responses, adaptive upregulation of PD-L1, increased IL-10 and TGF-β, re-emergence of Tregs and MDSCs, and re-establishment of suppressive TAM programs may collectively contribute to immunosuppressive rebound (6). Thus, PDT may transiently disrupt immune inertia, but it may also induce new forms of immunological negative feedback. The final immune outcome depends on treatment parameters, tumor type, and the timing of combination therapy.

### 5.1 TAM reprogramming: inflammatory activation and reparative rebound

TAMs represent a central hub in the remodeling of immunosuppressive networks after PDT. In the early phase, PDT-induced DAMPs, ROS, and inflammatory mediators may stimulate local macrophages to enhance phagocytosis, antigen processing, and the release of pro-inflammatory cytokines, thereby favoring the initiation of antitumor immunity (6). However, TAMs do not remain stably fixed in a pro-inflammatory state. As local hypoxia, clearance of necrotic tissue, and tissue-repair responses become more prominent, signals such as IL-10 and TGF-β may redirect macrophages toward immunosuppressive and tissue-repair-associated programs (17). This indicates that TAMs may contribute not only to early immune activation after PDT, but also to late therapeutic attenuation and immunosuppressive rebound.

### 5.2 Tregs and MDSCs: the cellular basis of post-treatment immune negative feedback

Regulatory T cells (Tregs) and myeloid-derived suppressor cells (MDSCs) represent another important set of factors limiting the sustained amplification of immunity after PDT. The early inflammatory response induced by PDT may alter the local myeloid-cell composition and transiently facilitate effector immune-cell entry into the tumor site. However, if antigen presentation and effector T-cell function are not sustained, Tregs and MDSCs may re-expand during the negative-feedback phase. Through inhibition of T-cell activation, depletion of local nutrients, secretion of immunosuppressive mediators, and reinforcement of immune-checkpoint signaling, these cells may weaken post-PDT antitumor immunity (33). Therefore, the rationale for combining PDT with Treg- or MDSC-modulating strategies is not merely to increase local tumor killing, but to prevent the post-treatment immune response from shifting back from inflammatory activation toward immunosuppression (34).

### 5.3 PD-1/PD-L1-mediated adaptive immune resistance

The PD-1/PD-L1 axis represents a key mechanistic node for combining PDT with immune checkpoint blockade. After PDT-induced antigen release, DC maturation, and T-cell infiltration, local interferon-related signaling may also promote adaptive upregulation of PD-L1, allowing tumor cells or myeloid cells to restrain effector T-cell function through the PD-1/PD-L1 axis. Therefore, combining PDT with PD-1/PD-L1 blockade has a strong mechanistic basis (7). In cSCC models, ALA-PDT combined with PD-L1 blockade has been reported to enhance antitumor effects against both primary and distant tumors and to improve the tumor immune microenvironment. These findings provide relatively direct preclinical support and translational rationale for combining PDT with immune checkpoint blockade in cutaneous tumors (35).

### 5.4 IL-10, TGF-β and hypoxia-driven suppressive feedback

IL-10, TGF-β and hypoxia represent key molecular components of suppressive feedback after PDT. High-light-dose PDT, particularly under high irradiance or insufficient local oxygenation, can induce rapid ROS generation, tumor microvascular injury and local oxygen depletion. Although these effects may contribute to acute tumor destruction, they may also promote tissue-repair programs and immunosuppressive feedback within the treated tumor microenvironment (1). Experimental evidence has shown that high-light-dose PDT can induce early immune activation followed by late-stage immunosuppression, with TGF-β1 playing an important role in this transition (6). Therefore, future optimization of PDT should not focus solely on increasing phototoxic injury, but should instead aim to prolong the immune-activation window while limiting delayed suppressive rebound.

Based on these mechanisms, the conversion of “cold” tumors into “hot” tumors is better understood as a conceptual framework for the immune-sensitizing potential of PDT, rather than as a uniform outcome after PDT in all cutaneous tumors. Under selected conditions, PDT may promote partial cold-to-hot immune remodeling through DAMP exposure, dendritic-cell maturation, activation of CXCL9/CXCL10-related chemokine axes (36), and increased CD8^+^ T-cell infiltration. However, this process may be incomplete or transient and can be constrained by PD-L1, TGF-β, Tregs/MDSCs, and hypoxia.

## 6 Influence of PDT parameters on the immune microenvironment

The immune effects of PDT are determined not only by tumor biology but also by the treatment regimen. Photosensitizer type and subcellular localization, drug–light interval, excitation wavelength, total light fluence, irradiance, tissue oxygenation, and treatment schedule may influence the site of ROS generation, the predominant mode of cell death, the profile and timing of DAMP emission, the extent of vascular injury, and the temporal window for immune-cell recruitment (37–39). Accordingly, PDT optimization should not be guided solely by the extent of local necrosis or immediate lesion clearance, but should also consider whether a given regimen promotes effective antigen presentation and a sustained antitumor immune response.

### 6.1 Photosensitizer type and subcellular localization

From the perspectives of clinical maturity and delivery format, photosensitizing approaches used or investigated in cutaneous tumor PDT can be broadly divided into three groups. The first comprises topically applied porphyrin-precursor prodrugs, primarily 5-aminolevulinic acid (5-ALA) and methyl aminolevulinate (MAL). Both enter the heme biosynthetic pathway and promote the intracellular accumulation of photoactive protoporphyrin IX (PpIX). ALA- and MAL-based PDT currently represent the most established dermatologic approaches for actinic keratoses, Bowen’s disease, superficial BCCs, and selected thin BCCs. The second group includes other exogenous photosensitizers, such as porphyrin derivatives, chlorins, and phthalocyanines, which may be administered systemically or formulated for alternative local delivery (1). Many of these agents exhibit red-shifted absorption and may offer greater tissue penetration, although their routine clinical role in cutaneous oncology remains limited. The third group comprises targeted or engineered photosensitizer platforms, including antibody- or ligand-conjugated photosensitizers, nanocarrier-based delivery systems, and organelle-targeted agents. These approaches are being developed mainly in preclinical models and combination-treatment settings (40). Consistent with this clinical hierarchy, the European Dermatology Forum guideline identifies topical PDT as an established treatment for actinic keratoses, Bowen’s disease, superficial BCCs, and selected thin BCCs (2).

The influence of photosensitizers on immune outcomes largely depends on their physicochemical properties and subcellular localization, which determine the primary site of ROS generation and thereby shape cellular stress responses, modes of cell death, and immunogenic output (1, 38). PDT targeting the endoplasmic reticulum or mitochondria is more likely to induce ER stress, mitochondrial injury, CRT exposure, and ATP or HMGB1 release, and may therefore be more readily coupled to ICD-associated signaling, DC maturation, and T-cell responses. By contrast, PDT preferentially affecting the plasma membrane or lysosomes may favor membrane damage, lysosomal dysfunction, or more lytic forms of cell death, which can generate strong inflammatory signals but do not necessarily translate into more efficient antigen presentation (38). Vascular-targeted PDT can rapidly reduce tumor burden through microvascular injury and perfusion shutdown; however, premature vascular shutdown may also reshape the temporal window for immune-cell entry and thereby limit sustained immune amplification (37).

When selecting a photosensitizing strategy in clinical practice, lesion depth, degree of hyperkeratosis, anatomical site, cosmetic requirements, recurrence risk and patient tolerability should be considered together. ALA/MAL-PDT is more suitable for superficial, low-risk, large-area lesions or lesions located in cosmetically sensitive areas. In contrast, hyperkeratotic lesions, nodular tumors, high-risk anatomical sites and recurrent lesions often require curettage, keratolytic treatment, laser pretreatment or other adjunctive approaches; when appropriate, treatment should be shifted to surgery, Mohs micrographic surgery, radiotherapy or systemic therapy according to tumor type and risk stratification (2, 41, 42). Current BCC guidelines emphasize that surgery remains the cornerstone of BCC management. Non-surgical approaches, including PDT, generally have lower cure rates than surgery and should mainly be considered for low-risk BCC or for patients in whom surgery is contraindicated (42).

### 6.2 Effects of light dose and illumination strategy

Illumination parameters are important upstream determinants of the immune effects of PDT. By influencing tissue penetration, PpIX photoactivation, oxygen consumption, vascular perfusion, and the temporal window for immune-cell recruitment, they shape the subsequent tumor immune microenvironment. Wavelength affects both photosensitizer excitation and tissue penetration. Blue light, typically within the range of 400–450 nm, penetrates relatively superficially and is therefore better suited to epidermal lesions such as actinic keratoses. By contrast, red light at approximately 630 nm penetrates more deeply and is more commonly used for relatively thicker but still superficial lesions, including Bowen’s disease and superficial basal cell carcinoma. Selection of the light source also depends on the photosensitizer formulation and clinical indication: ALA may be activated by either blue or red light, whereas MAL is commonly combined with red light (2, 43).

Total light fluence determines the cumulative intensity of photochemical injury, whereas irradiance primarily affects the rate of oxygen consumption and tissue oxygenation. Together, these parameters influence whether PDT remains within an immune-activating window or progresses more rapidly toward later immunosuppressive rebound. A higher total fluence can exacerbate cumulative tissue damage, while high irradiance may accelerate local oxygen depletion; when these effects produce intense photochemical stress, rapid ROS generation, vascular damage, and extensive necrosis may occur. Although such responses can provoke strong acute inflammation, they may also be accompanied by hypoxia, ischemia, and secondary immunosuppression. Experimental evidence indicates that high-light-dose PDT can induce early immune activation followed by late TGF-β1-associated immunosuppression. By contrast, lower irradiance or appropriately fractionated illumination may slow photochemical oxygen consumption and preserve local oxygenation, thereby providing a more favorable microenvironment for sustained danger-signal release, immune-cell recruitment, and effector function. Thus, illumination parameters determine not only the intensity of cell death but also the quality and durability of the subsequent immune response (6, 37).

In representative dermatologic PDT protocols, Bowen’s disease is commonly treated with red light irradiation at approximately 630 nm, and narrow-spectrum red light protocols frequently use a light fluence of approximately 37 J/cm². MAL-PDT is often administered as two treatment sessions separated by approximately 1 week (2). For studies aiming to exploit the immunosensitizing effects of PDT rather than merely enhancing local tumor ablation, lower light doses, lower irradiance and fractionated illumination have attracted increasing attention. These approaches are intended to slow photochemical oxygen consumption and maintain a more favorable local oxygenation state, thereby prolonging the immune-activation window (37, 44). Subtherapeutic photodynamic priming has mainly been explored as a strategy to improve tumor permeability and subsequent treatment delivery, while its immunomodulatory value remains investigational. Therefore, optimization of illumination parameters in cutaneous tumor PDT should balance sufficient immune activation, avoidance of excessive oxygen depletion, preservation of the immune-cell infiltration window and reduction of delayed suppressive feedback. It should not rely solely on increasing light fluence or irradiance to intensify the local treatment response. At present, whether low-dose, low-irradiance or fractionated illumination protocols can consistently improve the immune microenvironment after PDT in cutaneous tumors remains a parameter-optimization hypothesis requiring clinical validation.

### 6.3 Optimization of PDT treatment regimens

The core aim of PDT regimen optimization should not be limited to intensifying local phototoxic injury, but should instead focus on shaping a more exploitable immune-activation window. In addition to photosensitizer selection and illumination parameters, the drug–light interval or incubation time is also important, because it can influence the accumulation of photosensitizers or protoporphyrin IX (PpIX) within lesional tissue and alter the relative contribution of cellular versus vascular injury (1). A single PDT session can induce local photodynamic damage and short-term inflammation, whereas repeated treatment may theoretically prolong immune stimulation through recurrent tumor antigen release; however, whether this approach can consistently enhance immune memory remains to be verified (2). For superficial lesions with marked hyperkeratosis or heavy crusting, appropriate curettage, keratolytic debulking or laser pretreatment may improve photosensitizer penetration and effective illumination depth. Combination strategies should also consider treatment timing: mechanistically, immune checkpoint blockade may be combined with the early post-PDT window of inflammation, antigen release and T-cell recruitment, whereas interventions targeting suppressive axes such as TGF-β may be explored during the later negative-feedback phase (35).

Thus, the central challenge in PDT parameter optimization lies in balancing immune activation and tissue injury. Excessive phototoxicity may increase short-term tumor destruction and inflammatory intensity, but may also aggravate oxygen consumption, vascular shutdown and delayed immunosuppression. Conversely, insufficient treatment intensity may fail to generate effective DAMP exposure, tumor antigen release and local immune priming. Therefore, for PDT regimens designed for immunotherapy combinations, maximizing local necrosis should not be the sole objective. Instead, parameter design should aim to achieve sufficient antigen release, preserve local perfusion and the immune-cell infiltration window, and reduce delayed suppressive rebound.

## 7 Comparison of PDT-induced immune effects across different cutaneous tumors and precancerous lesions

Although PDT can contribute to antitumor immunity by inducing immunogenic cell death, releasing danger signals and reshaping the local inflammatory milieu, the baseline immune contexture, lesion depth and standard therapeutic pathways differ substantially across cutaneous tumors and precursor lesions. From the perspective of evidence maturity, PDT has a relatively well-established but selective clinical role in actinic keratosis, Bowen’s disease and superficial BCC (2). In invasive cSCC, its immunological relevance is better viewed as a potential local immune-priming or investigational combination strategy supported mainly by preclinical evidence, rather than as an established extension of routine clinical PDT indications (35). In melanoma, PDT remains largely within the domain of preclinical or research-oriented immunosensitization. Therefore, this section does not treat evidence from different cutaneous tumor types as equivalent. Instead, it compares the immunological implications, clinical boundaries and limits of extrapolation of PDT across squamous cell carcinoma-associated lesions, BCC and melanoma. Accordingly, the following comparison applies the evidence-maturity framework to distinguish established clinical indications from preclinical immune-combination evidence and exploratory immunosensitizing strategies.

### 7.1 The UV-associated keratinocyte neoplasia spectrum: AK, bowen’s disease, and cSCC

Actinic keratosis (AK), Bowen’s disease and invasive cutaneous squamous cell carcinoma (cSCC) can be discussed within the spectrum of UV-associated keratinocyte neoplasia. This spectrum reflects their shared association with cumulative ultraviolet damage, clonal keratinocyte alterations and field cancerization, but does not imply that all AK lesions inevitably or linearly progress to invasive cSCC (45). From the perspective of clinical evidence, the established use of PDT within this spectrum is concentrated mainly in AK and Bowen’s disease. AK can be treated with ALA/MAL-PDT or daylight PDT for both individual lesions and field cancerization, whereas Bowen’s disease is commonly treated with ALA- or MAL-mediated red-light PDT and represents one of the more established indications for topical PDT (2).

A multicenter randomized study of Bowen’s disease showed that MAL-PDT achieved a 12-month sustained complete response rate of 80%, compared with 67% for cryotherapy and 69% for 5-fluorouracil, with superior cosmetic outcomes (46). The advantages of PDT include tissue preservation, repeatability and suitability for multiple lesions or cosmetically sensitive sites. However, its efficacy is constrained by lesion thickness, degree of hyperkeratosis and photosensitizer penetration depth. For lesions suspicious for invasion or high-risk cSCC, treatment should still be guided by risk stratification and may require surgery, radiotherapy or systemic therapy; PDT should not be positioned as a substitute for standard curative treatment (41). Immunologically, ALA-PDT combined with PD-L1 blockade has shown potential to enhance control of both primary and distant tumors in cSCC models, providing preclinical support for PDT as a local immune-priming strategy, although this approach should not yet be regarded as an established clinical combination regimen (35).

### 7.2 Basal cell carcinoma

Topical PDT is a treatment option for superficial and selected low-risk thin nodular basal cell carcinomas (BCCs), particularly in patients for whom tissue preservation and cosmetic outcomes are major considerations. ALA- or MAL-mediated PDT is usually performed with red light irradiation at approximately 630 nm, and MAL-PDT is commonly administered as two treatment sessions separated by 1 week (2). However, complete surgical excision remains the first-line treatment for resectable BCC. For thicker nodular tumors, recurrent lesions, high-risk anatomical sites or aggressive histological subtypes, PDT is generally not preferred because its long-term local control is inferior to surgery (2, 42).

For locally advanced or metastatic BCC, Hedgehog pathway inhibitors remain important systemic treatment options; cemiplimab may be considered in patients whose disease has progressed after Hedgehog inhibitor therapy, who are intolerant to such treatment, or for whom Hedgehog pathway inhibition is inappropriate (42). Immunologically, Kabingu et al. showed that patients with BCC developed enhanced peripheral immune reactivity to the BCC-associated antigen Hip1 after PDT, providing human evidence that PDT can promote systemic recognition of tumor-associated antigens (18). However, this finding does not establish that PDT reduces recurrence risk. Therefore, the main value of PDT in BCC remains local control, tissue preservation and cosmetic benefit in low-risk lesions, rather than replacement of curative surgery.

### 7.3 Melanoma

Melanoma is a highly malignant and immunogenic cutaneous tumor, and immune checkpoint inhibitors have become a central component of treatment for high-risk resected and advanced melanoma (47). Unlike AK, Bowen’s disease and superficial BCC, conventional PDT has no established role as a standard primary treatment for melanoma. Its direct antitumor efficacy is constrained by lesion depth, tumor heterogeneity, melanin-related optical interference, antioxidant defenses and insufficient light penetration (48). These barriers are particularly relevant in pigmented melanoma. Melanin exhibits broad visible-light absorption that competes with photosensitizer excitation and reduces effective photon availability within pigmented lesions (49). Although photon propagation in melanoma is also influenced by tissue scattering, available optical measurements indicate that melanin itself contributes predominantly through absorption rather than acting as a major visible-wavelength scatterer (50). Melanin may additionally buffer photodynamic oxidative stress through its radical-scavenging and redox properties. This biochemical resistance is supported by experiments in human melanoma cells, in which pharmacological depigmentation increased PDT-induced intracellular ROS generation and enhanced susceptibility to photodynamic cell death (51). Thus, pigmentation may reduce PDT efficacy through both optical attenuation and redox buffering, in addition to heterogeneous photosensitizer accumulation, lesion depth and other tumor-intrinsic antioxidant defenses.

Despite this mechanistic rationale, clinical evidence for combining PDT with immune checkpoint blockade in cutaneous melanoma remains sparse. A targeted literature search did not identify published retrospective clinical cohorts evaluating PDT–ICI combinations specifically in cutaneous melanoma. Current evidence remains predominantly preclinical, including B16-F10 melanoma studies in which PDT combined with a flagellin-adjuvanted cancer vaccine potentiated anti-PD-1-mediated tumor control (52). Consequently, the clinical efficacy, optimal treatment sequence and safety profile of PDT–ICI combinations in melanoma cannot yet be established without extrapolation from ICI-only cohorts or non-cutaneous PDT–ICI studies.

Accordingly, there is no mature or unified conventional PDT parameter regimen for melanoma. Current research has mainly focused on modified photosensitizers, nanodelivery systems, near-infrared or two-photon strategies, and immunotherapy combinations. The translational significance of PDT in melanoma therefore lies not in routine local ablation, but in its potential use as a local immune-priming approach that induces immunogenic tumor injury, antigen release and inflammatory microenvironmental remodeling. Such strategies remain investigational and should not yet be regarded as established clinical treatment modalities (48, 52).

## 8 PDT-based combination immunotherapy: from local photodynamic injury to an immunotherapeutic window

From an immunotherapeutic perspective, the rationale for combining PDT with other immune interventions is not simply to intensify local tumor destruction, but to exploit the transient immune window created by photodynamic injury. PDT can promote tumor antigen release, DAMP exposure, innate immune recruitment, dendritic-cell activation, and subsequent T-cell priming, thereby providing a biological basis for combination with immune-checkpoint blockade, myeloid-cell modulation, innate immune agonists, or vaccine-based strategies (7). The success of such combinations depends on whether the selected tumor type and PDT regimen generate sufficient immunogenic input, whether the partner therapy targets a relevant post-PDT immune bottleneck, and whether the timing of intervention matches the kinetics of immune activation and suppressive feedback (6, 35).

### 8.1 PDT combined with PD-1/PD-L1 blockade

PD-1/PD-L1 blockade represents one of the most mechanistically plausible directions for combining PDT with immunotherapy. After PDT-induced antigen release, dendritic-cell activation and T-cell recruitment, local immune visibility may be increased. At the same time, inflammation-related signals may also drive adaptive upregulation of PD-L1 on tumor cells or myeloid cells, thereby limiting effector T-cell function. Therefore, combining PD-1/PD-L1 blockade within the post-PDT immune-activation window may help counteract adaptive immune resistance (7). In a cSCC model, ALA-PDT combined with PD-L1 blockade enhanced antitumor effects against both primary and distant tumors and optimized the tumor immune microenvironment (35). These findings provide preclinical support for combining PDT with immune checkpoint blockade in cutaneous tumors. Future studies should further define the temporal window of PD-L1 induction, the quality of T-cell infiltration and whether distant-tumor responses can serve as clinically relevant translational endpoints.

### 8.2 Timing of combination therapy

The efficacy of combining PDT with immune checkpoint inhibitors (ICIs) depends not only on whether the two modalities are combined, but also on treatment timing. PDT-induced acute inflammation may enhance antigen presentation, T-cell priming and tumor infiltration, whereas T-cell maturation and activation may lag behind local photodynamic injury. In a preclinical study, Wu et al. showed that T-cell maturation and activation lagged approximately 3–7 days behind PDT, and that the timing of ICI administration affected the efficacy of combination therapy (53). In parallel, high-light-dose PDT may be followed by late-stage immunosuppressive feedback associated with TGF-β1 (6). Future translational studies should therefore compare concurrent administration, short-delay ICI administration after PDT and multi-cycle combination regimens, rather than relying solely on empirical combination designs.

Human longitudinal immunomonitoring data in cutaneous tumors remain limited. In patients with superficial BCC, serial biopsies obtained before and at multiple time points up to 72 h after ALA-PDT demonstrated time-dependent changes in apoptosis and inflammatory-cell composition, providing direct evidence that the post-PDT immune microenvironment evolves dynamically in humans (11). However, this study evaluated PDT alone rather than its combination with immune checkpoint blockade. Outside cutaneous oncology, retrospective clinical data in gastric cancer, together with serial pre- and post-PDT immune profiling, provide human proof-of-principle that PDT can remodel immune-cell infiltration and T-cell clonality in patients receiving checkpoint immunotherapy (54). Nevertheless, in our targeted supplementary search, we did not identify published multicenter cohorts with longitudinal immune profiling specifically evaluating PDT–ICI combinations in cutaneous tumors. Accordingly, the optimal timing of PDT and checkpoint blockade in cutaneous oncology is still supported predominantly by preclinical kinetics and requires prospective clinical validation.

Clinical safety data specific to cutaneous PDT–ICI combinations are similarly insufficient. ICIs can induce a broad spectrum of cutaneous immune-related adverse events, including macular or eczematous eruptions, pruritus, psoriasis and other immune-mediated inflammatory dermatoses (55). By contrast, conventional dermatologic PDT more commonly produces localized pain, burning, erythema and other treatment-site reactions (56). These represent clinically and mechanistically distinct toxicity patterns, and it remains unknown whether preceding or concurrent PDT alters the incidence, phenotype or severity of ICI-associated cutaneous immune toxicity. Future PDT–ICI trials should therefore prospectively document the onset, morphology and grade of cutaneous adverse events and distinguish expected local phototoxic reactions from systemic or immune-mediated toxicity.

### 8.3 PDT combined with Treg/MDSC modulation

Tregs, MDSCs and suppressive TAM programs may contribute to the attenuation of post-PDT immune effects. During the early phase after PDT, DAMP exposure and inflammatory signaling can increase local immune visibility. However, the subsequent accumulation of suppressive lymphoid or myeloid populations may constrain effector T-cell function (33). Therefore, the rationale for combining PDT with Treg/MDSC modulation is not to intensify phototoxic injury, but to maintain the post-PDT inflammatory and antigen-presenting state and reduce its transition toward suppressive feedback. Preclinical studies have shown that Treg depletion can enhance PDT-mediated antitumor immunity (57), and high-light-dose PDT may also be followed by a late immunosuppressive phase associated with TGF-β1 (6). At present, these strategies remain mainly within preclinical or mechanistic research, and cutaneous tumor-specific clinical evidence remains insufficient.

### 8.4 PDT combined with TLR agonists or tumor vaccines

PDT can provide tumor antigens and DAMP-associated danger signals, but antigen release alone does not necessarily translate into effective T-cell priming. Additional immunoadjuvant signals delivered through TLR agonists or tumor vaccine platforms may further promote dendritic-cell maturation, migration to draining lymph nodes and CD8^+^ T-cell activation (7). In a melanoma model, PDT combined with a flagellin-adjuvanted cancer vaccine potentiated anti-PD-1-mediated tumor suppression, suggesting that PDT-induced local tumor injury can be coupled with vaccine-like immune amplification strategies (52). However, cutaneous tumor-specific evidence remains limited, and these approaches should currently be regarded as experimental immune-amplification strategies rather than established clinical combination regimens.

### 8.5 PDT combined with novel photosensitizers and nanoplatforms

Novel photosensitizers and nanoplatforms may improve tumor selectivity, photosensitizer delivery, tissue penetration, oxygenation control and subcellular localization, thereby expanding the design space for PDT-based immunotherapy combinations. These platforms can also be integrated with immune checkpoint blockade, immunoadjuvants or regulated cell death-modulating strategies to enhance immunogenic tumor injury (7). However, most nanoplatform-based studies remain confined to preclinical models. The induction of ICD, inflammatory cell death or checkpoint sensitization in animal experiments should not be directly equated with clinical evidence in cutaneous tumors. At present, these platforms mainly provide evidence for mechanistic extension and delivery optimization. Their clinical translational value will depend on formulation reproducibility, scalable manufacturing, standardization of illumination parameters and appropriate matching with cutaneous tumor indications (58). Within the evidence-maturity framework, most nanoplatform-based immune-sensitizing strategies should therefore be positioned as Level 3 exploratory evidence, or at most Level 2 evidence when supported by cutaneous tumor-relevant animal or ex vivo validation.

## 9 Discussion: limitations, future directions and conclusions

### 9.1 Limitations of current evidence

Although PDT-mediated remodeling of the immune microenvironment in cutaneous tumors is biologically plausible, the current evidence remains uneven across tumor types, experimental systems and levels of clinical maturity. Established clinical evidence is concentrated mainly in actinic keratosis, Bowen’s disease, superficial BCC and selected low-risk thin BCC, whereas many immune-combination concepts in cSCC and melanoma are still supported primarily by preclinical or exploratory data. This distinction is central to the argument of this review, because clinical indications, immune mechanistic plausibility and nanoplatform-based experimental findings do not carry the same evidentiary weight.

A major limitation is that many findings regarding ICD, regulated cell death, TAM reprogramming and immune checkpoint sensitization are derived from non-cutaneous tumor models, *in vitro* systems or engineered nanoplatforms (7). These studies are valuable for identifying mechanistic possibilities, but they often lack direct validation in human cutaneous tumor tissues or under clinically relevant PDT conditions. This limitation is particularly relevant for pyroptosis, necroptosis, ferroptosis, cuproptosis and nanoplatform-based immune-sensitizing strategies, where current evidence is stronger for biological plausibility than for disease-specific clinical translation.

PDT parameter heterogeneity represents another source of uncertainty. Across studies, photosensitizer formulation, incubation time, wavelength, light fluence, irradiance, tissue oxygenation, illumination mode and treatment schedule vary substantially (2). These variables can influence ROS localization, DAMP emission, vascular injury, immune-cell recruitment and delayed suppressive feedback. As a result, immune outcomes observed under one PDT regimen may not be directly comparable with those generated under another. This heterogeneity also complicates efforts to define an optimal immune-activation window for combination therapy.

Clinical immunomonitoring remains insufficiently standardized. Representative immunopharmacodynamic readouts, including CD8^+^ T-cell infiltration, dendritic-cell activation, antigen-presentation markers, PD-L1 expression, Tregs/MDSCs, macrophage states, CXCL9/CXCL10-related chemokine axes, IFN-related signatures and TGF-β/IL-10-associated suppressive signals, are not consistently incorporated into clinical PDT studies. Consequently, it remains difficult to determine whether PDT induces only a transient inflammatory response, a sustained antitumor immune response or a clinically actionable immune-combination window. The evidence-maturity framework used in this review highlights this gap by separating established clinical indications from cutaneous tumor-specific translational studies and nanoplatform-based exploratory mechanisms.

### 9.2 Strengths and limitations of this review

This narrative review has several limitations inherent to its design. A strength of the review is its explicit evidence-maturity framework, which was used to distinguish established clinical applications from cutaneous tumor-relevant translational evidence and exploratory mechanistic studies. Although PubMed, Web of Science Core Collection and Google Scholar were searched using predefined thematic blocks, the review was not designed as a systematic review. Accordingly, study identification and evidence appraisal were narrative rather than exhaustive, and no PRISMA-based systematic screening, formal risk-of-bias assessment or quantitative synthesis was undertaken. Initial screening by one reviewer, followed by targeted verification of key studies and boundary cases by a second reviewer, may have introduced reviewer-dependent selection bias. The prioritization of English-language publications may also have resulted in language bias and the omission of relevant evidence published in other languages. In addition, substantial heterogeneity across the primary literature—including photosensitizer formulation, ALA versus MAL, incubation or drug–light interval, blue versus red illumination, fluence, irradiance, lesion depth, treatment schedule and immune-assessment time points—limits direct comparison across studies (2). Less frequently investigated immune-cell populations may also have been underrepresented in the initial search strategy; a targeted supplementary search was therefore performed for mast-cell-related evidence.

### 9.3 Future directions and translational priorities

Future research should move beyond lesion clearance alone and define the immune-activation window, suppressive feedback and targetable intervention points after PDT. The translational question is therefore more specific: which tumor types, PDT regimens and combination schedules can produce immune effects that are durable enough to influence clinical outcomes? Photosensitizer localization, light fluence, irradiance, tissue oxygenation and treatment frequency may jointly shape ICD quality, DAMP exposure, antigen presentation, immune-cell recruitment and subsequent immunosuppression.

A more clinically useful next step is to establish tumor type-specific immune-monitoring strategies. For AK, Bowen’s disease and low-risk BCC, future work may clarify how topical PDT reshapes field cancerization, local immune surveillance and recurrence-related immune features. For cSCC, studies should determine whether PDT can reproducibly create a local immune-priming state that improves the efficacy of immune checkpoint blockade or other immunomodulatory interventions. For melanoma, the field should remain focused on preclinical and translational validation of photosensitizer modification, optical optimization and immunotherapy combinations, rather than positioning conventional PDT as a routine local treatment.

Treatment timing also requires more systematic evaluation. This is particularly relevant when PDT is combined with immune checkpoint blockade, Treg/MDSC modulation, TGF-β/IL-10-axis intervention, TLR agonists or tumor vaccines. Future translational studies should compare concurrent administration, delayed administration and multi-cycle combination regimens while integrating tumor-type stratification and immune monitoring (6, 53). Ideally, combination timing should be aligned with the kinetics of antigen release, dendritic-cell activation, T-cell priming, PD-L1 induction and delayed suppressive feedback.

For nanoplatforms and novel photosensitizers, translational discipline should become a central design criterion. Future studies should prioritize formulation reproducibility, scalable manufacturing, clinically feasible illumination parameters, safety and disease-specific validation in cutaneous tumor models. Nanoplatforms that induce ICD, inflammatory cell death or checkpoint sensitization in animal experiments should be interpreted as exploratory or translational tools unless they are supported by cutaneous tumor-specific validation and clinically relevant treatment conditions.

Overall, PDT should no longer be viewed merely as a local phototoxic treatment. In AK, Bowen’s disease and superficial or selected low-risk BCC, PDT already has a defined role in local control, field cancerization management, tissue preservation and cosmetic benefit. In cSCC, its value is better positioned in the treatment of precursor or *in situ* lesions and as a local immune-priming strategy for exploratory immunotherapy combinations. In melanoma, conventional PDT remains mainly within the research domain of novel photosensitizers, delivery systems and immunotherapy-combination platforms. The central value of PDT in cutaneous tumor immunotherapy lies in its capacity to induce parameter-tunable ROS-related injury, ICD, DAMP release, innate immune-cell recruitment and dendritic-cell-mediated antigen presentation, thereby providing a translational entry point for local control and immune-combination strategies in selected cutaneous tumors. Its clinical value will ultimately depend on standardized PDT parameters, tumor type-specific evidence and prospective immune-monitoring studies.
